# Antenatal corticosteroids: a reappraisal of the drug formulation and dose

**DOI:** 10.1038/s41390-020-01249-w

**Published:** 2020-11-11

**Authors:** Alan H. Jobe, Matthew Kemp, Augusto Schmidt, Tsukasa Takahashi, John Newnham, Mark Milad

**Affiliations:** 1 Division of Obstetrics and Gynecology, The University of Western Australia, Perth, WA Australia; 2 Perinatal Institute, Department of Pediatrics, Cincinnati Children’s Hospital Medical Center, University of Cincinnati, Cincinnati, OH USA; 3 Centre for Perinatal and Neonatal Medicine, Tohoku University Hospital, Sendai, Japan; 4 School of Veterinary and Life Sciences, Murdoch University, Perth, WA Australia; 5 Division of Neonatology, Department of Pediatrics, University of Miami, Miami, FL USA; 6 Milad Pharmaceutical Consulting, Plymouth, MI USA

## Abstract

**Abstract:**

We review the history of antenatal corticosteroid therapy (ACS) and present recent experimental data to demonstrate that this, one of the pillars of perinatal care, has been inadequately evaluated to minimize fetal exposure to these powerful medications. There have been concerns since 1972 that fetal exposures to ACS convey risk. However, this developmental modulator, with its multiple widespread biologic effects, has not been evaluated for drug choice, dose, or duration of treatment, despite over 30 randomized trials. The treatment used in the United States is two intramuscular doses of a mixture of 6 mg betamethasone phosphate (Beta P) and 6 mg betamethasone acetate (Beta Ac). To optimize outcomes with ACS, the goal should be to minimize fetal drug exposure. We have determined that the minimum exposure needed for fetal lung maturation in sheep, monkeys, and humans (based on published cord blood corticosteroid concentrations) is about 1 ng/ml for a 48-h continuous exposure, far lower than the concentration reached by the current dosing. Because the slowly released Beta Ac results in prolonged fetal exposure, a drug containing Beta Ac is not ideal for ACS use.

**Impact:**

- Using sheep and monkey models, we have defined the minimum corticosteroid exposure for a fetal lung maturation.
- These results should generate new clinical trials of antenatal corticosteroids (ACS) at much lower fetal exposures to ACS, possibly given orally, with fewer risks for the fetus.

## Current treatments with antenatal corticosteroids (ACS)

ACS treatments are standard treatments for all women at risk of preterm delivery worldwide as recommended by obstetric practice guidelines^1^ and the World Health Organization (WHO) as the most effective way to decrease preterm infant deaths.^2^ As such, one may ask why should we be re-evaluating that treatment? As stated in 2015 in the J*ournal of the American Medical Association*, relative to the Declaration of Helsinki:

Even the best proven interventions must be evaluated continually through research for their safety, effectiveness, efficacy, accessibility and quality.^3^

ACS is a unique therapy for a very high-risk population (the preterm fetus) developed initially by Liggins and Howie in 1972 ^4^ based on results from sheep models from the 1960s.^5^ It is surprising that the initial treatment of two 12 mg doses of a 1:1 mixture of betamethasone phosphate (Beta P) and betamethasone acetate (Beta Ac) proposed and tested by Liggins in his 1972 randomized controlled trial remains the standard therapy in the US, Europe, and Australia.^6^ Liggins^5^ selected the mixture because the slowly released Beta Ac would accomplish longer fetal exposures. However, the need for a prolonged fetal exposure was never verified as essential for the clinical response until we tested the Beta Ac component of the two-drug combination used in clinical practice in sheep and monkey models recently.^7–16^ Most of the repeat treatment regimens that are used worldwide have not been tested for safety and efficacy.

**Fig. 1:**
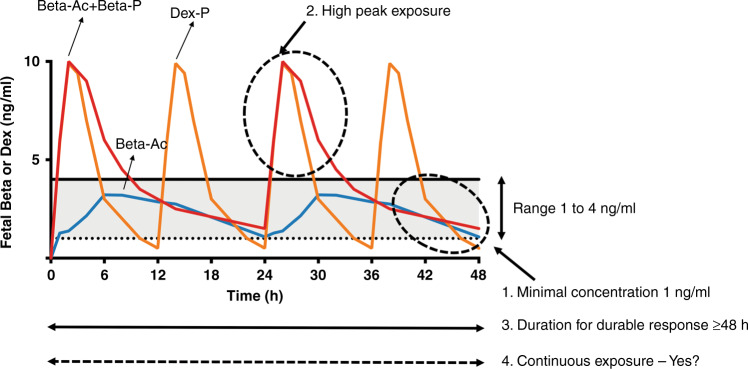
Sketch of fetal blood levels estimated from cord blood.^45^ These curves represent predicted fetal plasma after maternal treatments with the 3 mg betamethasone phosphate (Beta P) plus 3 mg betamethasone acetate (Beta Ac) mixture given 24 h apart (red curve), 3 mg dexamethasone phosphate (Dex P) given every 12 h (orange curve), and 3 mg Beta Ac component (blue curve) based on measurements in a sheep model with the characteristics of drug levels that we will discuss. The light gray area on the figure is our estimate of a good target for fetal plasma levels for lung maturation. Issues for dosing are: 1. The range for minimal dose for fetal lung response. 2. Need for high peak exposure. 3. Nature of exposure—duration and continuous for a durable response. Redrawn from Ballard and Ballard.^45^

The current WHO recommended treatment is four doses of 6 mg dexamethasone phosphate (Dex P) (total dose 24 mg) because of low costs and widespread availability.^2^ A recent international multicenter randomized controlled trial (RCT) compared two doses of 12 mg Beta P and Beta Ac against two doses of 12 mg Dex P spaced by 24 h had equivalent outcomes, demonstrating that the drugs are interchangeable for the ACS indication.^17^ In a recent Cochrane review of ACS, other treatments that have been tested clinically were reviewed.^18^ Multiple aspects of ACS dosing have not been systematically evaluated, including which drug, what treatment interval, which dose, and which drug for which treatment interval. The basic pharmacokinetics (PK) of the major drugs used for ACS have been minimally studied in pregnant women and their fetuses.^19^ The PK and pharmacodynamics (PD) are complex as a drug is given to the pregnant woman to treat her fetus. This creates multiple variables of maternal PK, placental drug transfer and metabolism, blood levels in the fetus and fetal metabolism, as well as drug response levels for the multiple PD effects in the human fetus, which can only be evaluated after delivery using clinical outcomes and drug levels in cord blood (Fig. 1). For example, we do not know if the same exposure will benefit lung maturation, death, and intraventricular hemorrhage (IVH) or does each benefit require a different dose and period of exposure. We will use only lung maturation (lung compliance and gas exchange after preterm delivery) as the measurement for corticosteroid PD effect for this review as there is more information from RCTs about lung maturation than other effects.

## Concerns about current dosing

1. The standard dose of 24 mg Dex P or Beta P + Beta Ac is a very large dose of corticosteroids based on common medical usage.^20^
2. Corticosteroids are potent modulators of development and there are indications that there may be effects on human populations from ACS including birth weight^21^ and neurodevelopment.^22,23^ Risks are proportional to dose.^24^
3. Larger numbers of fetuses are being exposed beyond the initial indication of 24–34 weeks gestational age with the anticipation of imminent preterm deliveries for late preterm deliveries,^25^ very preterm, periviable deliveries, and elective C-sections.^26^
4. There is no information that ACS actually benefit pregnancies in low and middle income countries (LMIC) where most of the premature deaths occur.^27^ In fact, newborn mortality increased with ACS use in environments with marginal medical care for the women and the infant following preterm delivery at 24–34 weeks.^28^
5. Treatments that contain Beta Ac result in very long fetal exposures if the fetus does not deliver within 4–7 days of the efficacy interval for minimal benefit.^2^
6. The many meta-analyses of ACS simply evaluate small variances of the Liggins dosing. The large number of trials have hidden the fact that no dose ranging information is available for ACS.
7. There has been no input from regulatory agencies for ACS therapy and it is not FDA approved. Many clinical research resources were used that did not add new information.^29^

The meta-analyses are extensive and simply represent small variances of the Liggins dosing with no attempt to do formal dose range testing to define a minimal dose to limit fetal exposure and complications.^6,18,30,31^ As all the dosing is high, the trials comparing drugs are not helpful for minimizing dosing and defining minimal exposures. From our perspective, the number of trials and total number of patients enrolled with consistent outcomes has hidden the fact that no dose ranging information is available for ACS to evaluate effectiveness after treatment, perhaps the most tested intervention in perinatal medicine.

## PK in nonpregnant women

**Fig. 2:**
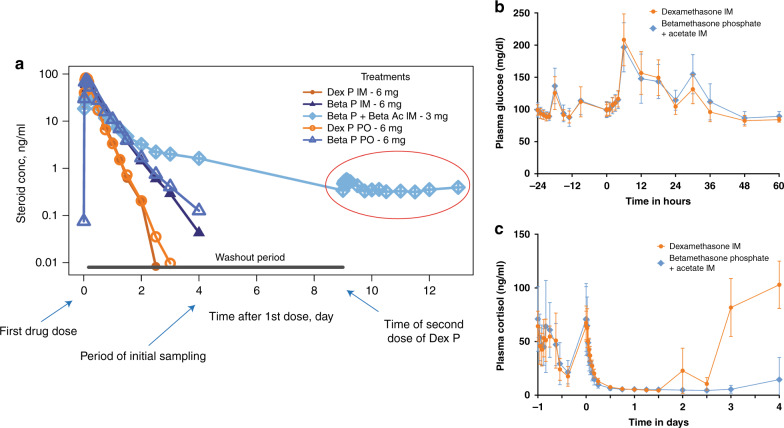
Pharmacokinetic and pharmacodynamics studies of corticosteroids in nonpregnant, reproductive age women. **a** Pharmacokinetic measurements of betamethasone phosphate (Beta Phos—6 mg) given orally or IM, dexamethasone (Dex Phos—6 mg) given orally or IM dose, or 6 mg of the 1:1 mixture of Beta Phos (3 mg) plus Beta Acetate (Beta Ac, 3 mg) given IM to healthy Indian women with blood measurements over 96 h and after 9 days from the initial dose (redrawn from ref. ^34^). Beta from the Beta P plus Beta Ac had a slow clearance with blood levels measurable 9 days after the initial dose and with no further apparent clearance between 9 and 13 days from the Beta Ac component. **b** Effects of ACS on plasma glucose. A single dose of 6 mg dexamethasone P or the 1:1 mixture of 3 mg Beta P + 3 mg of Beta Ac increased post-prandial blood glucose. All PO and IM treatments had comparable effects on blood sugar (redrawn from ref. ^34^). **c** Effects of single ACS doses on cortisol. A single dose of 6 mg dexamethasone P or the 1:1 mixture of 3 mg Beta P + 3 mg of Beta Ac suppressed cortisol, but the Beta P plus Beta Ac treated group had longer suppression of cortisol to more than 4 days (redrawn from ref. ^34^).

Beta P and Dex P are rapidly dephosphorylated to betamethasone or dexamethasone within minutes^32^ to yield the nonphosphorylated betamethasone or dexamethasone. The ratio of maternal Beta to fetal Beta is about 0.4 using cord blood at the time of delivery if delivery is within 6 days of treatment.^33^ Therefore, shortly after a maternal 12 mg dose, maternal Beta levels should be about 100 ng/ml from the 6 mg Beta P component of the drug^34^ (Fig. 2). This is a high level relative to the much lower level that is needed for lung maturation, as we will develop below.

We recently reported a large PK/PD study in 48 normal weight reproductive age nonpregnant women from Bangalore, India who received one 6 mg dose of Beta P or Dex P PO or IM (Fig. 2).^34^ With samples from 12 or 24 women for each treatment of either 6 mg of Beta P + Beta Ac, 6 mg of Beta P, or 6 mg of Dex P, the absorption and clearance curves were very consistent (Fig. 2a). Both Beta P and Dex P were rapidly absorbed (1.5 h oral, 3 h IM) with comparable maximal concentrations (65–95 ng/ml) for IM or oral dosing. In contrast, the maximal concentration for the IM Beta P + Beta Ac mixture was about 50% of the level for the phosphorylated drug because the slowly absorbed Beta Ac did not contribute to the initial blood levels at early times. The plasma half-life of dexamethasone (5.2 h) was cleared twice as fast as for betamethasone (*t*_1/2_ 10.2 h) when each were given IM or PO. We treated the women with 1 drug and after a washout period of 9 days after the first drug, we administered a second drug (Dex P, PO, or IM) and the corticosteroid concentrations were again measured to 96 h. At 9 days after Beta P + Beta Ac, the betamethasone concentrations was 0.36 ± 0.88 ng/ml. This would translate to 1.36 ng/ml for a 12 mg exposure to Beta Ac for the clinical treatment which contains 12 mg Beta Ac. Consistent with an NICHD data set of women treated with 12 mg Beta P + Beta Ac had maternal plasma levels of about 3 ng/ml at 6.5 days after the second 12 mg drug dose.^33^ A caution of interpretation: blood concentrations are likely to be different in pregnant women because of multiple pregnancy-related metabolic changes, but there is not good data of high resolution in pregnancy.^33^

Fetal cord blood levels 6.5 days after the last treatment were about 2 ng/ml.^33^ In a recent second report of betamethasone concentrations after the two-dose clinical treatment,^35^ cord blood betamethasone concentrations after maternal treatment measured in cord blood found maternal concentrations of about 4 ng/ml at 3–4 days after the second dose and cord blood concentrations also at over 4 ng/ml.^35^ The maternal/fetal blood concentration ratio was reported to be about 0.4,^29,35^ but that ratio changes clearly. Maternal and cord blood levels were comparable in maternal plasma and cord plasma at a later time after treatment by 3–4 days,^33,35^ probably because the betamethasone levels had reached an equilibrium between maternal and fetal compartments.

Although we are focusing on lung maturation effects, corticosteroids have pleotropic effects on the mother and fetus. From our Bangalore study of PK in nonpregnant healthy women, we also measured PD effects on glucose and cortisol and other white blood cells—all were significantly altered by 6 mg doses of Beta P or Dex P (Fig. 2b, c).^34^

Baseline plasma glucose values for the 24 h before corticosteroid treatment were very similar across the five treatment groups (Fig. 2a).^34^ Blood glucose increased similarly in the treatment groups from the fasting baseline of about 100 mg/dl to about 200 mg/dl in association with lunch after an overnight fast. Thus, a single dose of 6 mg Beta or Dex had a large effect on maternal plasma glucose for 24 h with residual effects into day 2, when, in clinical practice, pregnant women would be receiving a second dose, which would further alter blood sugar.

The average AM plasma cortisol was about 70 ng/ml with a normal diurnal rhythm prior to treatment (Fig. 2c).^34^ The five-drug treatments caused rapid and similar decreased cortisol to a minimal concentration of about 4 ng/ml with similar periods of suppression for about 60 h for oral and IM Dex P. Cortisol was suppressed for about 72 h for the oral or IM Beta P. The Beta P + Beta Ac caused a prolonged suppression for more than 4 days.^34^ Assuming routine clinical treatment with two doses of Beta Ac + Beta P, the adrenal suppression could be much longer, so that second course of treatment may keep the pregnant woman immunosuppressed for several weeks.

Therefore the PK measurements give us two valuable pieces of information: (1) Dex P has about a twofold shorter half-life compared to Beta P, which will influence the number and interval of doses used for modeling dosing for both Dex P and Beta P (Fig. 1).^36^ (2) The information suggests the potential to decrease risks by using lower doses that Beta P + Beta Ac poses for neurodevelopmental injury^37^ is the persistence of betamethasone in cord blood for 1 week after treatment.^37^ The combination drug is a poor choice for a fetal therapy as the standard treatment is exposing the fetus to measurable blood concentrations for at least two weeks. This observation results in our statement that because of the prolonged fetal exposures we have been using the wrong drug for 48 years. The IM Beta Ac forms a delayed release Beta source after IM injection that results in very slow drug release.

The use of a drug with Beta Ac also makes no sense in terms of the large literature and discussions of repeated courses of ACS. All the studies of repeated courses of ACS used Beta P + Beta Ac for the initial treatment.^23^ The standard treatment has a treatment effect that probably lasts 2 weeks already; thus, there should be no need for repeated treatments. The benefits of repeated treatments are quite modest in a recent individual participant meta-analysis,^23^ which does not surprise us in light of the pharmacokinetic data. A complete unknown is the fate of maternally derived Dex or Beta in the fetus after delivery.

## What minimal Beta exposure is required for lung maturation in sheep?

In 2007, we published an experiment where pregnant ewes received one dose of 0.5 mg/kg Beta P or a single dose of 0.25 mg/kg Beta P or one or two doses maternal IM Beta P + Beta Ac mixture (0.5 mg/kg).^14^ We found that one dose of 0.5 mg/kg IM Beta P did not cause lung maturation but the Beta P + Beta Ac combination did cause lung maturation. All treatments would have caused high initial maternal plasma levels^34^ for a treatment to delivery interval of 2 days. Despite these very high doses of the Beta P, there was no lung maturation. Therefore, the high peak maternal blood concentrations that occur soon after treatment are not sufficient by themselves to cause the lung maturation response.

**Fig. 3:**
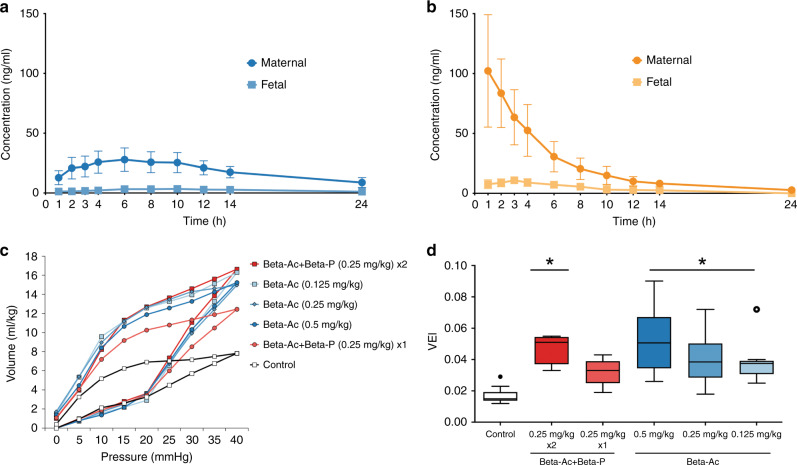
Testing for minimal ACS dose for fetal lung maturation in sheep. **a** Maternal and fetal plasma levels of betamethasone for treatments with 0.125 mg/kg Beta Ac maternal IM. Area under the curve (AUC_0–24 h_) is 443 ng/ml h for Beta in the ewe and 56 ng/ml h in the fetus. The fetal levels stay above 1 ng/ml for 24 h. Redrawn from ref. ^16^ **b** Maternal and fetal drug levels after 0.125 mg/kg Beta P. AUC_0–24_ in ewes were 557 and 79 ng/ml h in fetus. Redrawn from ref. ^16^ The fetal levels remain above 1 ng/ml for only 8 h despite the very high plasma levels in the ewes. **c** Pressure–volume curves after preterm delivery and ventilation measured 2 days after a single treatment, showing similar improvements of the low-dose Beta Ac and the clinical dosing. **d** The composite gas exchange variable, Ventilation Efficiency Index (VEI) which incorporates tidal volume/kg, peak ventilation pressure, PaCO_2_, and ventilation rate for the clinical dosing and Beta Ac component alone showing similar improvements of the VEI with low-dose Beta Ac and the clinical dosing. Redrawn from ref. ^16^. *Different than control *p* < 0.05.

In 2009,^15^ we made extensive PK and PD measurements for the drugs used for ACS with support from the Bill & Melinda Gates Foundation^22^ (Fig. 3). Using the same sheep model as in 2007, we treated pregnant ewes with 0.5, 0.25, or 0.125 mg/kg Beta Ac only in comparison to one or two doses of Beta Ac + Beta P—the clinical combination drug. In this experiment we tested Beta Ac at a lower dose and did more precise maternal and fetal blood level measurements (Fig. 3). Two days after the initial treatment, a single dose of 0.125 mg/kg of Beta Ac was as effective in inducing lung maturation as two doses of 0.25 mg/kg Beta P + Beta Ac. So in sheep we estimate a minimal fetal exposure to Beta to be about 2 ng/ml based on the PK results for Beta Ac (Fig. 3a). The clinical dose is 24 mg. If we assume that the average women in preterm labor weighs 70 kg, then her corticosteroid exposure is 0.34 mg/kg. In clinical practice, ACS corticosteroid dose has not been adjusted for maternal weight or body mass index,^25,38^ and clinical outcomes have been insensitive to maternal weight or multiple fetuses —probably because the dose treatments are already so high.

## Beta P infusions to test minimum exposure and durability of lung maturation—sheep

**Fig. 4:**
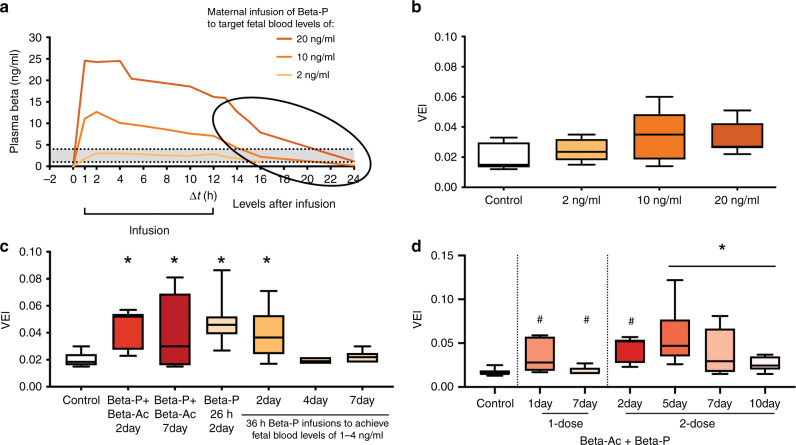
Minimal dose and durability in sheep. **a** Constant betamethasone infusions in pregnant ewes to target fetal plasma levels of 20 (maternal infusions of Beta P loading dose 0.28 ng/kg then 0.48 ng/kg/12 h), 10 (loading dose of 0.14 ng/kg + 0.24 ng/kg over 12 h), or 2 ng/ml (loading dose of 0.028 ng Beta P + 0.048 ng/kg/12 h infusion), with documentation of fetal levels of Beta for 24 h. **b** Evaluation of lung performance by measuring VEI after infusion to the three target levels.^7^ **c** Additional data for fetuses infused with Beta P to achieve a fetal exposure of 1 ng/ml for 36 h. There was no durable effect at 4 or 7 days. The clinical treatment requires two doses for durability in sheep. Redrawn from ref. ^7^ **d** Ewes were treated with one or two doses of IM 0.25 mg/kg Beta P plus Beta Ac. The lambs were delivered at intervals from treatment of 1–10 days a single dose improved preterm lung function at 1 day but not 7 days. The two-dose treatments had efficacy to 7 days with residual effects at 10 days.

A more revealing approach was evaluated in sheep using maternal infusions of Beta P to achieve target blood levels of betamethasone in the fetus (Fig. 4a).^7^ We used loading doses and constant maternal infusions of Beta P to target stable fetal blood levels of 20, 10, or 2 ng/ml for 12 h. This PK study demonstrated that fetal blood levels much higher than the target range were achieved for 12 h for the 20 ng/ml group with rapid clearance from the fetal circulation after 12 h (Fig. 4a). Figure 4 has a gray zone from 1 to 4 ng/ml fetal betamethasone because fetal betamethasone levels in that range for 24 h were effective for lung maturation using Beta Ac in sheep.^16^

To evaluate lung function, separate groups of pregnant ewes were given the same targeted infusions and delivered 48 h after the initiation of the infusions and ventilated for 30 min. The values at 30 min for the Ventilation Efficacy Index demonstrated that the 20 ng/ml exposure induced lung maturation, while the 10 ng/ml had inconsistent results (Fig. 4b) and the 2 ng/ml target was not effective (Fig. 4). Despite the 10 ng/ml plasma target infusion greatly exceeding the exposure from 0.125 mg/kg Beta Ac for 12 h, there was minimal lung maturation suggesting that a 12 h exposure was insufficient to induce lung maturation. The static lung gas volumes at necropsy gave the same pattern of results. However, subsequent clearances were sufficiently slow to keep Beta levels above 1 ng/ml for about 24 h for those animals in the 20 ng/ml target group (Fig. 4a) and lung maturation occurred in the animals. The 2 ng/ml target was below 1 ng/ml by 16 h and no maturation occurred. Based on the result with the 10 ng/ml group and the efficacy of 0.125 mg/kg Beta Ac, a treatment interval of <24 h is not sufficient to induce lung maturation.

We also tested the durability of 1 vs 2 doses of Beta Ac + Beta P—the clinical dose in sheep. One dose induced lung maturation at 24 h and 48 h (Fig. 4c), but that effect was lost by 7 days (Fig. 4d). In contrast, two doses at a 24-h interval had a durable effect to 7 days with some residual effects at a 10-day treatment to delivery interval.^9^ These PK data were generated with the maternal infusions of Beta P and maternal IM Beta P + Beta Ac. The fetal exposure profile for optimal responses at low total dose should be generally applicable for any route of treatment or corticosteroid, adapted to maternal–fetal transfer and potency of the corticosteroid. The loss of effects after the 36 h infusions at 4 and 7 days gave us the first indication that there was a problem with durability of ACS effects on the fetal lung (Fig. 4c).

Clinical information about durability of ACS has not been carefully explored. The WHO reanalysis^2^ of the Roberts meta-analysis found that for each benefit,^6^ the effective interval from treatment to delivery differed (e.g. deaths decreased for intervals <48 h only) while respiratory distress syndrom (RDS) decreased from 48 h to 7 days but not after 7 days and IVH decreased only for an interval >48 h. Liebowitz and Clyman^39^ reported increased IVH for an interval from treatment of >10 days and Ring et al.^40^ reported an increase risk of ventilator support for fetuses exposed to ACS at <28 weeks who delivered at >28 weeks. The long intervals after ACS have not been considered as an important variable in response and are difficult to study in clinical cohorts unless there are time-matched control groups,^19^ but this question can be studied with some precision in large animal models. The biology about how a maturational signal to mature a fetal organ which than can reverse to immaturity remains to be explained, but is an important consideration for treatment strategies.

## Minimal exposure for lung maturation in monkeys

**Fig. 5:**
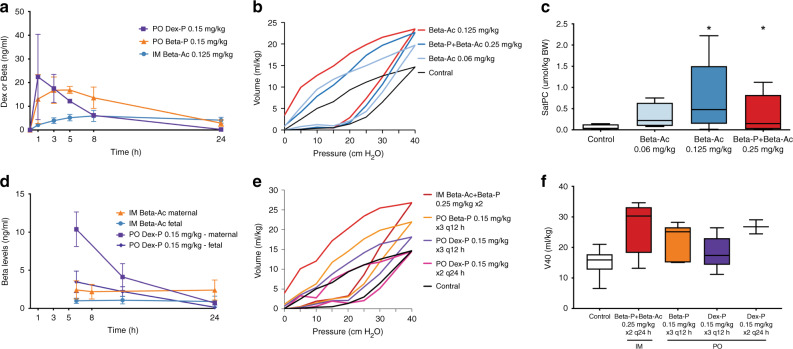
Minimal corticosteroid dose for lung maturation in monkeys. **a** PK data for 0.125 mg/kg Beta Ac maternal IM or 0.15 mg/kg oral Dex P from nonpregnant monkeys. Redrawn from ref. ^8^ **b** Fetal monkeys at about 30 days gestational age were treated with maternal Beta Ac given IM 0.125 mg/kg and 5 days later were delivered for measurement of static pressure–volume curves (redrawn from ref. ^12^). **c** Sat phosphotidylcholine in alveolar wash was also used as a maturation marker which increased with the low-dose Beta Ac treatment. **d** PK data for 0.125 mg/kg Beta Ac maternal IM or 0.15 mg/kg oral Dex P from monkey fetuses. The curves have only three data points because samples from fetuses were collected with ultrasound-guided chordocentesis. The low dose of 0.125 mg/kg yielded fetal plasma levels of about 1 ng/ml for 24 h. equivalent to the much higher exposure with clinical dose (redrawn from ref. ^12^). **e**, **f** Fetal monkeys at about 30 days gestational age were treated with maternal oral Beta P or Dex P and 5 days later were delivered for measurement of static pressure–volume curves. Redrawn from refs. ^8,12^ *Different from control (*p* < 0.05).

A parallel experiment in the pregnant Rhesus macaque using ultrasound-guided fetal blood sampling demonstrated similar low peak fetal blood betamethasone concentrations of about 5 ng/ml for maternal doses of 0.125 mg/kg Beta Ac. This was as effective as 0.25 mg/kg of Beta P + Beta Ac for fetal lung maturation and increased surfactant in alveolar wash, but achieved very low fetal blood concentrations of about 1 ng/ml at 48 h for a maturational assessment at 5 days^8^ (Fig. 5c). Dosing monkeys with a lower dose of 0.06 mg/kg Beta Ac did not induce lung maturation (Fig. 5b), suggesting we have identified an effective low fetal concentration of >1 ng/ml in fetal plasma.^8^

## Effects on the fetal primate brain

**Fig. 6:**
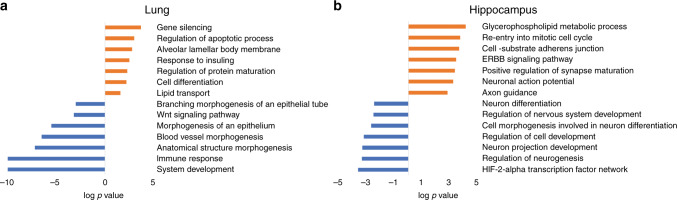
Lung and hippocampus RNA-sequencing after treatment with ACS, studied 5 h after the ACS dose or saline control (*n* = 3 animals/group). **a** Gene set enrichment analysis for biological processes associated with differentially regulated genes in the fetal lung 5 h after treatment with Beta Ac evaluated 5 days after treatment. **a** ACS treatment induced cellular differentiation and surfactant-related processes as well as apoptosis while suppressing immune response and lung developmental pathways. **b** Biological processes altered associated with differentially regulated genes in the fetal hippocampus for 5 h after 0.125 mg/kg Beta Ac +  Beta P resulted in suppression of genes associated with neuronal cell differentiation and neurogenesis. All redrawn from ref. ^12^

Transcriptome analysis of the fetal lung and brain after treatment with ACS has provided insight on the pathways regulated by ACS in these organs and potential side effects (Fig. 6). While 0.125 mg/kg Beta Ac + Beta P induced genes associated with lung cellular differentiation and surfactant production, it also caused suppression of lung morphogenesis pathways and induction of apoptosis-related genes even after a short 5 h fetal exposure to low-dose Beta Ac.^12^ In the fetal hippocampus ACS also had unintended signaling consequences with suppression of neuronal developmental pathways.^12^ These findings identify the pleiotropic effects of ACS and potential toxicity of these drugs to fetal lung and brain development.

## Oral dosing with a continuous exposure above the response threshold is necessary

We have identified an approximate response threshold for fetal plasma concentration of >1 ng/ml in sheep and monkeys and the inference of a similar value for the humans.^33,35^ That threshold is easily achieved using the prolonged and continuous exposure from Beta Ac with the two doses of the clinical drug. The requirement for a continuous exposure above a threshold for 48 h has not been formally tested by prospectively designed experiments. However, in the monkey, 0.15 mg/kg of oral Dex P given at 0 and 24 h intervals did not improve lung gas volumes measured at 40 cm H_2_O pressure (V_40_).^8^ Oral Beta P given as 0.15 mg/kg as three doses at 12 h intervals did increase V_40_ (Fig. 5e). These results in monkeys are consistent with the need for a continuous exposure resulting from the longer half-life of Beta than Dex in fetal plasma (Fig. 4e). Our initial results with two doses of Beta P or Dex P in sheep also indicate a continuous exposure is required for good maturational responses.^11^ Our results with oral dosing in sheep further indicate that continuous exposures for 48 h are required.^13^

## Summary

The PK information is of high resolution with the sheep and with the Indian women because of the larger number of observations that were possible and the ability to assess PD at intervals from 1 to 10 days after dosing in the sheep. We only evaluated the fetal monkeys 5 days after treatment initiation. The results are internally consistent for the two animal models. As the half-life of betamethasone is twice as long as for dexamethasone, Beta P can be dosed less frequently than Dex P to keep the concentration above 1 ng/ml in the fetus. Oral and IM dosing PK information was very similar for each treatment, when the oral dose was given to the fasted women. The Beta P + Beta Ac mixture had a very slow clearance because of the delayed release of betamethasone from the acetate prodrug. Of note, the one 3 mg dose of Beta Ac used for our measurements in humans was 25% that used clinically, and betamethasone could be measured 2 weeks and would presumably be 4× higher with the two-dose clinical treatment (12 mg) (Fig. 2).^34^ We view this persistence beyond the exposure interval needed for lung maturation to be undesirable for mother and fetus.

## ACS and personalized medicine

Personalized medicine is an attempt to selectively treat patients who will benefit from the treatment and not be harmed by other outcomes which are a risk from ACS. ACS in RCTs decrease RDS by only 40%.^2^ The majority of women at risk of preterm delivery and treated with ACS do not benefit from ACS, which means their fetuses are unnecessarily exposed to corticosteroids. The benefits for late preterm infants are modest as are benefits for elective C-sections.^26^ A poorly documented number of women have threatened preterm labor and are given ACS and deliver at term with some clinical data suggesting adverse effects on fetal growth and neurodevelopmental outcomes.^21,37,41^ There is also no RCT data showing benefits for periviable deliveries prior to 24 weeks.^19^

Therefore, our approximate estimate is that 40–50% of all deliveries in the US may receive ACS which benefit only a minority of the pregnancies with a dose of ACS that is the wrong drug at too high a dose.^9^ Fetal lung maturation-related biomarkers can be measured using mRNA in amniotic fluid^42^ as can biomarkers for gestation age in maternal blood.^43^ In the future, a test for fetal lung maturity using maternal blood^43^ could allow the clinician to only treat appropriate infants who would get RDS. Ideally, with lower more appropriate doses, only fetuses who will benefit from ACS will be exposed to ACS.

Such considerations may be more important in low resource environments where many at risk patients may respond differently and have different risks treatments with ACS may increase infant mortality^28^ and corticosteroid induced hypoglycemia in the infant may not be recognized and effectively treated.^44^ There is no dose response information about ACS effects on other fetal organs. We used lung function as it can be comparably measured across species with studies. Dosing schedules to test low-dose strategies have been reported.^35,36^ These low-dose strategies need to be tested in randomized controlled trials.

Our conclusions are:

1. High fetal plasma levels do not contribute to the fetal lung responses and are undesirable because of potential toxicity.
2. A threshold fetal plasma concentration for fetal maturation is about 1 ng/ml for Beta or Dex for 48 h. The threshold exposure of 1 ng/ml is an estimate made with some precision using perfusion models (only for Beta P) and the depo form of Beta Ac in sheep, and with less precision range fetal plasma levels in monkeys, and estimates of exposures in humans from cord blood.^20,33,35^ Results using oral dosing also support this approximate value in sheep and monkeys. Thus, the conclusion that standard treatment is using the concentration of >1 ng/ml is adequate for lung maturation. The current clinical dosing results in much higher concentrations.
3. The duration of exposure is critical for a durable response—that duration of exposure is ≥48 h in the sheep. Clinical data also support that duration of exposure is important for clinical outcomes.^2^ The Beta Ac component of the clinical drug will accomplish that, but two doses are required for a durable response in sheep (Fig. 4). The downside of Beta Ac is the prolonged maternal and fetal exposure if delivery occurs at >48 h after treatment.
4. Oral or IM Dex P or Beta P will cause comparable lung maturation if fetal exposures are comparable,^8,13^ based on dosing to compensate for the PK differences in clearance of the two drugs.
5. There seems to be no substantial difference in maturation response with Dex or Beta so that either drug can be used for ACS.^17^
6. Even the very low fetal exposures alter the transcriptome to indicate potential adversity of the fetal lung and brain within 5 h.^12^

## Gaps in knowledge

- Prove with an equivalency clinical trial that low-dose ACS (IM or oral) are comparable to present standard treatments.
- Test efficacy and risks of ACS with clinical studies in LMIC environments.
- Consider dosing based on maternal weight.
- Understand where ACS signaling occurs in the fetal lung—to possibly develop a treatment with less pleotropic effects for antenatal treatments.
- Develop new fetal evaluations that could be used to identify fetuses that may benefit from ACS.
- Explain the loss of durable lung maturation with time after treatment.

## References

[1] Committee Opinion No. (2017). 713 summary: antenatal corticosteroid therapy for fetal maturation. Obstet. Gynecol..

[2] *WHO Recommendations on Interventions to Improve Preterm Birth Outcomes* (WHO Guidelines Approved by the Guidelines Review Committee, Geneva, 2015). 26447264

[3] World Medical A. (2013). World Medical Association Declaration of Helsinki: ethical principles for medical research involving human subjects. JAMA.

[4] Liggins GC, Howie RN (1972). A controlled trial of antepartum glucocorticoid treatment for prevention of the respiratory distress syndrome in premature infants. Pediatrics.

[5] Liggins GC (1969). Premature delivery of foetal lambs infused with glucocorticoids. J. Endocrinol..

[6] Roberts D, Brown J, Medley N, Dalziel SR (2017). Antenatal corticosteroids for accelerating fetal lung maturation for women at risk of preterm birth. Cochrane Database Syst. Rev..

[7] Kemp MW (2018). The efficacy of antenatal steroid therapy is dependent on the duration of low-concentration fetal exposure: evidence from a sheep model of pregnancy. Am. J. Obstet. Gynecol..

[8] Schmidt AF (2019). Oral dosing for antenatal corticosteroids in the Rhesus macaque. PLoS ONE.

[9] Kemp MW (2020). The duration of fetal antenatal steroid exposure determines the durability of preterm ovine lung maturation. Am. J. Obstet. Gynecol..

[10] Kemp MW (2016). Maternofetal pharmacokinetics and fetal lung responses in chronically catheterized sheep receiving constant, low-dose infusions of betamethasone phosphate. Am. J. Obstet. Gynecol..

[11] Schmidt AF (2017). Antenatal dexamethasone vs. betamethasone dosing for lung maturation in fetal sheep. Pediatr. Res..

[12] Schmidt AF (2019). Dosing and formulation of antenatal corticosteroids for fetal lung maturation and gene expression in rhesus macaques. Sci. Rep..

[13] Schmidt AF (2019). Oral antenatal corticosteroids evaluated in fetal sheep. Pediatr. Res..

[14] Jobe AH (2007). Betamethasone for lung maturation: testing dose and formulation in fetal sheep. Am. J. Obstet. Gynecol..

[15] Jobe AH (2009). Betamethasone dose and formulation for induced lung maturation in fetal sheep. Am. J. Obstet. Gynecol..

[16] Schmidt AF (2018). Low-dose betamethasone-acetate for fetal lung maturation in preterm sheep. Am. J. Obstet. Gynecol..

[17] Crowther CA (2019). Maternal intramuscular dexamethasone versus betamethasone before preterm birth (ASTEROID): a multicentre, double-blind, randomised controlled trial. Lancet Child Adolesc. Health.

[18] Brownfoot F. C., Gagliardi D. I., Bain E., Middleton P. & Crowther C. A. Different corticosteroids and regimens for accelerating fetal lung maturation for women at risk of preterm birth. *Cochrane Database Syst Rev*. 2013: CD006764. 10.1002/14651858.CD006764.pub3 23990333

[19] Jobe AH, Goldenberg RL (2018). Antenatal corticosteroids: an assessment of anticipated benefits and potential risks. Am. J. Obstet. Gynecol..

[20] Waljee AK (2017). Short term use of oral corticosteroids and related harms among adults in the United States: population based cohort study. BMJ.

[21] Rodriguez A (2019). Antenatal corticosteroid therapy (ACT) and size at birth: a population-based analysis using the Finnish Medical Birth Register. PLoS Med..

[22] Jobe AH (2020). Antenatal corticosteroids—a concern for lifelong outcomes. J. Pediatr..

[23] Crowther CA (2019). Effects of repeat prenatal corticosteroids given to women at risk of preterm birth: an individual participant data meta-analysis. PLoS Med..

[24] Sarnes E (2011). Incidence and US costs of corticosteroid-associated adverse events: a systematic literature review. Clin. Ther..

[25] Gyamfi-Bannerman C (2016). Antenatal betamethasone for women at risk for late preterm delivery. N. Engl. J. Med..

[26] Saccone G, Berghella V (2016). Antenatal corticosteroids for maturity of term or near term fetuses: systematic review and meta-analysis of randomized controlled trials. BMJ.

[27] Althabe F (2015). A population-based, multifaceted strategy to implement antenatal corticosteroid treatment versus standard care for the reduction of neonatal mortality due to preterm birth in low-income and middle-income countries: the ACT cluster-randomised trial. Lancet.

[28] Althabe F (2016). The Antenatal Corticosteroids Trial (ACT)‘s explanations for neonatal mortality—a secondary analysis. Reprod. Health.

[29] Sinclair JC (1995). Meta-analysis of randomized controlled trials of antenatal corticosteroid for the prevention of respiratory distress syndrome: discussion. Am. J. Obstet. Gynecol..

[30] Crowley PA (1995). Antenatal corticosteroid therapy: a meta-analysis of the randomized trials, 1972 to 1994. Am. J. Obstet. Gynecol..

[31] Roberts, D. & Dalziel, S. Antenatal corticosteroids for accelerating fetal lung maturation for women at risk of preterm birth. *Cochrane Database Syst. Rev*. 2006, CD004454. 10.1002/14651858.CD004454.pub2 16856047

[32] Samtani MN, Schwab M, Nathanielsz PW, Jusko WJ (2004). Area/moment and compartmental modeling of pharmacokinetics during pregnancy: applications to maternal/fetal exposures to corticosteroids in sheep and rats. Pharm. Res..

[33] Gyamfi C (2010). The effect of plurality and obesity on betamethasone concentrations in women at risk for preterm delivery. Am. J. Obstet. Gynecol..

[34] Jobe AH, Milad MA, Peppard T, Jusko WJ (2020). Pharmacokinetics and pharmacodynamics of intramuscular and oral betamethasone and dexamethasone in reproductive age women in India. Clin. Transl. Sci..

[35] Foissac F (2020). Maternal betamethasone for prevention of respiratory distress syndrome in neonates: population pharmacokinetic and pharmacodynamic approach. Clin. Pharm. Ther..

[36] Ke AB, Milad MA (2019). Evaluation of maternal drug exposure following the administration of antenatal corticosteroids during late pregnancy using physiologically-based pharmacokinetic modeling. Clin. Pharm. Ther..

[37] Raikkonen K, Gissler M, Kajantie E (2020). Associations between maternal antenatal corticosteroid treatment and mental and behavioral disorders in children. JAMA.

[38] Hashima JN (2010). The effect of maternal body mass index on neonatal outcome in women receiving a single course of antenatal corticosteroids. Am. J. Obstet. Gynecol..

[39] Liebowitz M, Clyman RI (2016). Antenatal betamethasone: a prolonged time interval from administration to delivery is associated with an increased incidence of severe intraventricular hemorrhage in infants born before 28 weeks gestation. J. Pediatr..

[40] Ring AM (2007). The effect of a prolonged time interval between antenatal corticosteroid administration and delivery on outcomes in preterm neonates: a cohort study. Am. J. Obstet. Gynecol..

[41] Paules C (2017). Threatened preterm labor is a risk factor for impaired cognitive development in early childhood. Am. J. Obstet. Gynecol..

[42] Kamath-Rayne BD (2015). Systems biology evaluation of cell-free amniotic fluid transcriptome of term and preterm infants to detect fetal maturity. BMC Med. Genomics..

[43] Ngo TTM (2018). Noninvasive blood tests for fetal development predict gestational age and preterm delivery. Science.

[44] Kamath-Rayne BD, Rozance PJ, Goldenberg RL, Jobe AH (2016). Antenatal corticosteroids beyond 34 weeks gestation: what do we do now?. Am. J. Obstet. Gynecol..

[45] Ballard PL, Ballard RA (1995). Scientific basis and therapeutic regimens for use of antenatal glucocorticoids. Am. J. Obstet. Gynecol..

